# Local overexpression of Su(H)-MAPK variants affects Notch target gene expression and adult phenotypes in *Drosophila*

**DOI:** 10.1016/j.dib.2015.11.004

**Published:** 2015-11-10

**Authors:** Jasmin S. Auer, Anja C. Nagel, Adriana Schulz, Vanessa Wahl, Anette Preiss

**Affiliations:** Universität Hohenheim, Insitut für Genetik (240A), Garbenstr. 30, 70599 Stuttgart, Germany

**Keywords:** *Drosophila*, EGFR signalling, MAPK, Notch signalling, Su(H)

## Abstract

In *Drosophila*, Notch and EGFR signalling pathways are closely intertwined. Their relationship is mostly antagonistic, and may in part be based on the phosphorylation of the Notch signal transducer Suppressor of Hairless [Su(H)] by MAPK. Su(H) is a transcription factor that together with several cofactors regulates the expression of Notch target genes.

Here we address the consequences of a local induction of three *Su*(*H*) variants on Notch target gene expression. To this end, wild-type *Su*(*H*), a phospho-deficient *Su*(*H*)^*MAPK-*^^*ko*^ and a phospho-mimetic *Su*(*H*)^*MAPK-ac*^ isoform were overexpressed in the central domain of the wing anlagen. The expression of the Notch target genes *cut, wingless, E*(*spl*)*m8-HLH* and *vestigial*, was monitored. For the latter two, reporter genes were used (*E*(*spl*)*m8-lacZ, vg*^*BE*^*-lacZ*). In general, *Su*(*H*)^*MAPK-*^^*ko*^ induced a stronger response than wild-type *Su*(*H*), whereas the response to *Su*(*H*)^*MAPK-ac*^ was very weak. Notch target genes *cut, wingless* and *vg*^*BE*^*-lacZ* were ectopically activated, whereas *E*(*spl*)*m8-lacZ* was repressed by overexpression of Su(H) proteins. In addition, in epistasis experiments an activated form of the EGF-receptor (*DER*^*act*^) or the MAPK (*rl*^*SEM*^) and individual Su(H) variants were co-overexpressed locally, to compare the resultant phenotypes in adult flies (thorax, wings and eyes) as well as to assay the response of the Notch target gene *cut* in cell clones.

Specifications Table

**Table:** 

Subject area	*Biochemistry, Genetics and Molecular Biology*
More specific subject area	*Developmental Biology, Cellular signalling*
Type of data	*Figures and text*
How data was acquired	*Microscopy: Zeiss Axioskop linked to a Bio-Rad MRC1024 confocal microscope; Wild 5M stereomicroscope and Zeiss Axiophot coupled to an Optronics ES120 camera*
Data format	*Filtered data, analyzed*
Experimental factors	*Imaginal discs were dissected, fixed, washed and blocked before adding antibodies.*
Experimental features	*Tissue-specific expression of respective transgenes was induced with the Gal4::UAS-system. Gene expression was monitored directly or from reporter genes by antibody staining of the protein products.*
Data source location	*n.a.*
Data accessibility	*The data is with this article*

Value of the data

• This data shows the responses of several Notch target genes to modulations of Su(H) activity by the EGFR pathway.
• The data allow for a visual comparison of the spectrum of Notch target gene responses to Su(H) overexpression.
• Overexpression of activated components of the EGFR pathway and Su(H) variants, alone or in combination, can be compared in various *Drosophila* tissues.
• This data may be extended by analyses on *DER*^*act*^ activity during *Drosophila* wing development.

## 1 Data

Suppressor of Hairless [Su(H)] is the transcription factor that regulates the expression of the target genes of the Notch signalling pathway [1], [2]. Su(H) protein may be phosphorylated by MAPK as a result of Epidermal Growth Factor Receptor (EGFR) activation, providing a means of a direct cross-talk between these two pathways [3], [4], [5]. The response of several Notch target genes to the modulations of Su(H) by EGFR signalling activity was analysed by the local overexpression of either wild-type *Su*(*H*), a phospho-deficient *Su*(*H*)^*MAPK-*^^*ko*^ and a phospho-mimetic *Su*(*H*)^*MAPK-ac*^ variant [3] using the Gal4::UAS system [6], and staining of the tissues with respective antibodies. Moreover, activated components of the EGFR pathway (*DER*^*act*^, *rl*^*SEM*^) were overexpressed alone or in combination with individual *Su*(*H*) variants. The response of the Notch target gene *cut* was observed in cell clones of wing imaginal discs, and the resultant phenotypes on thorax, wings and eyes were recorded in adult flies.

### 1.1 Overexpression of *Su*(*H*) variants during wing development

**Fig. 1:**
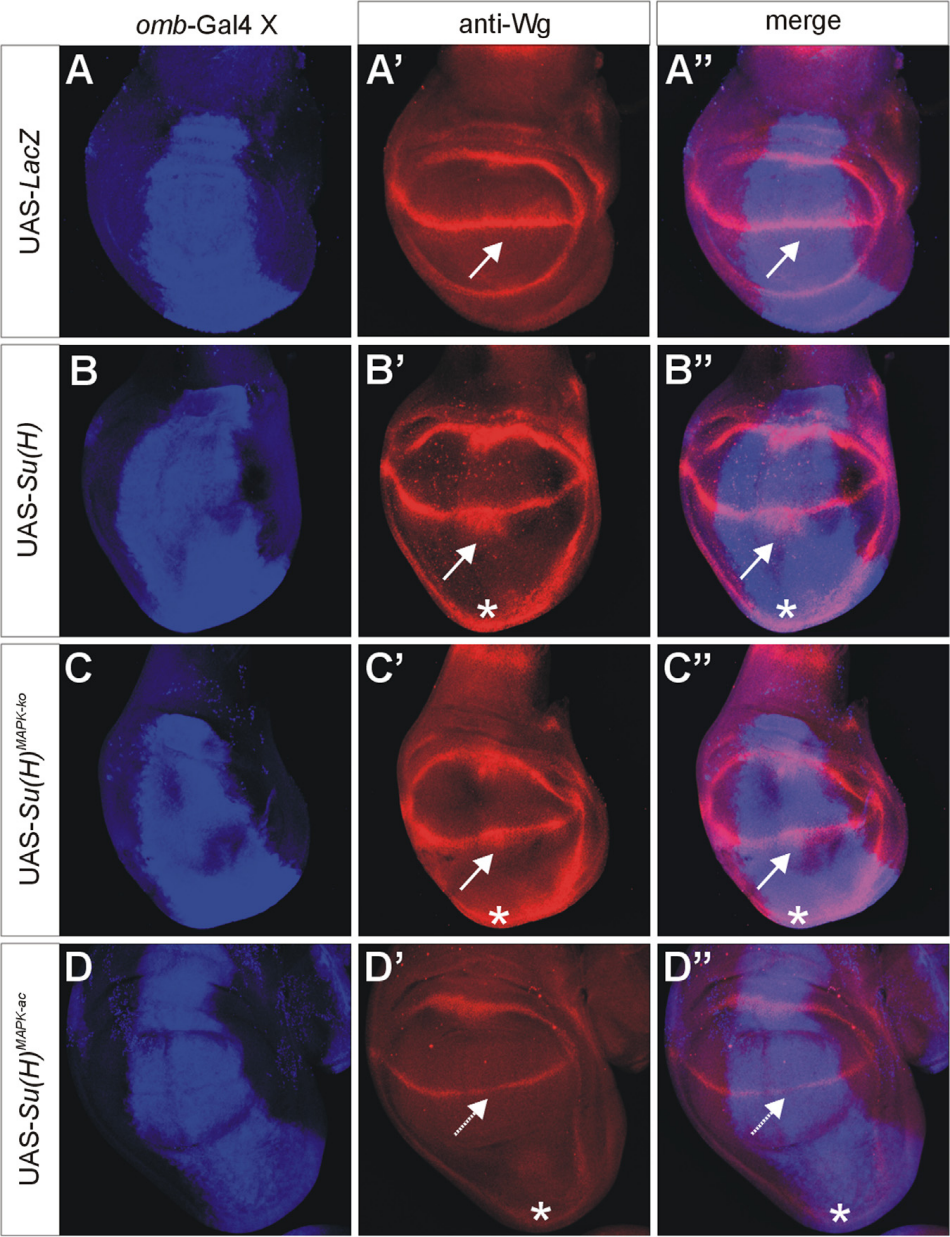
Response of the Notch target gene *wingless.* Overexpression of the UAS-*Su*(*H*) variants as indicated; the *omb*-expression domain is highlighted in blue in A–D and A′′–D″ (A,A′ anti-beta galactosidase staining; B–D and B′–D′, anti-Su(H) staining). Expression of *wingless* (Wg) is shown in red (A′–D″). UAS-*lacZ* served as control. Note expansion of *wingless* expression along the dorso-ventral boundary (arrows) upon overexpression of *Su*(*H*) and *Su*(*H*)^*MAPK-*^^*ko*^, but not *Su*(*H*)^*MAPK-ac*^. Overgrowth of the ventral disc is marked by asterisks and is a consequence of the overexpression of Su(H) protein (B′–D″).

**Fig. 2:**
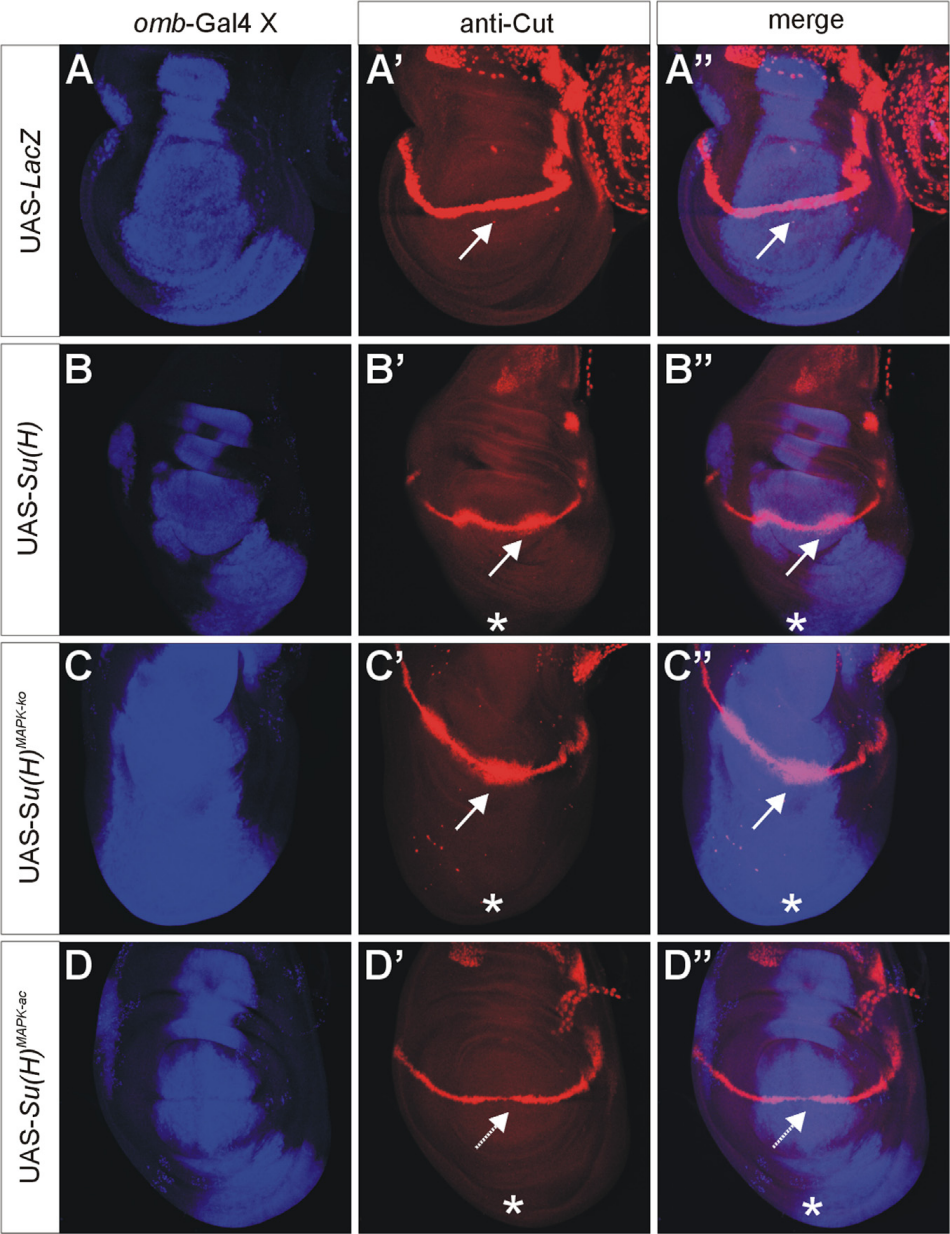
Response of the Notch target gene *cut*. Overexpression of the UAS-*Su*(*H*) variants as indicated; the *omb*-expression domain is highlighted in blue in A–D and A″–D″ (A,A′ anti-beta galactosidase staining; B–D and B′–D′, anti-Su(H) staining). Expression of *cut* is shown in red (A′–D″). UAS-*lacZ* served as control. Note expansion of *cut* expression along the dorso-ventral boundary (arrows) upon overexpression of *Su*(*H*) and *Su*(*H*)^*MAPK-*^*^ko^*, but not *Su*(*H*)^*MAPK-ac*^. Overgrowth of the ventral disc is marked by asterisks and is a consequence of the overexpression of Su(H) protein (B′–D″).

**Fig. 3:**
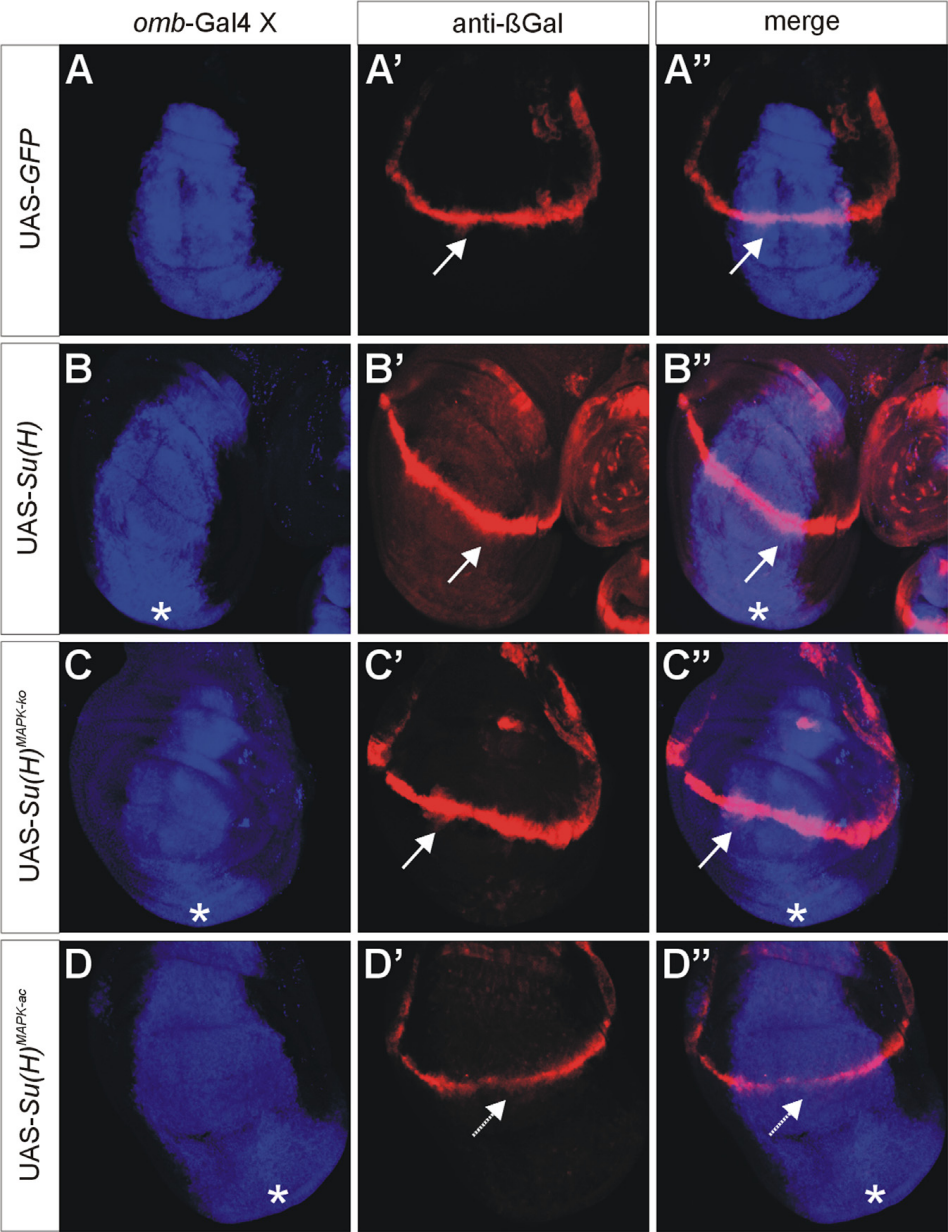
Response of the Notch target gene *vestigial*. Overexpression of the UAS-*Su*(*H*) variants as indicated; the *omb*-expression domain is highlighted in blue in A–D and A″–D″ (A,A′ green fluorescent protein GFP; B–D and B′–D′, anti-Su(H) staining). Expression of the *vestigial* reporter *vg*^*BE*^*-lacZ* is shown in red (A′–D″). UAS-*GFP* served as control. Note expansion of *vg*^*BE*^*-lacZ* expression along the dorso-ventral boundary (arrows) upon overexpression of *Su*(*H*) and *Su*(*H*)^*MAPK-*^*^ko^*, but not *Su*(*H*)^*MAPK-ac*^. Overgrowth of the ventral disc is marked by asterisks and is a consequence of the overexpression of Su(H) protein (B–D, B″–D″).

**Fig. 4:**
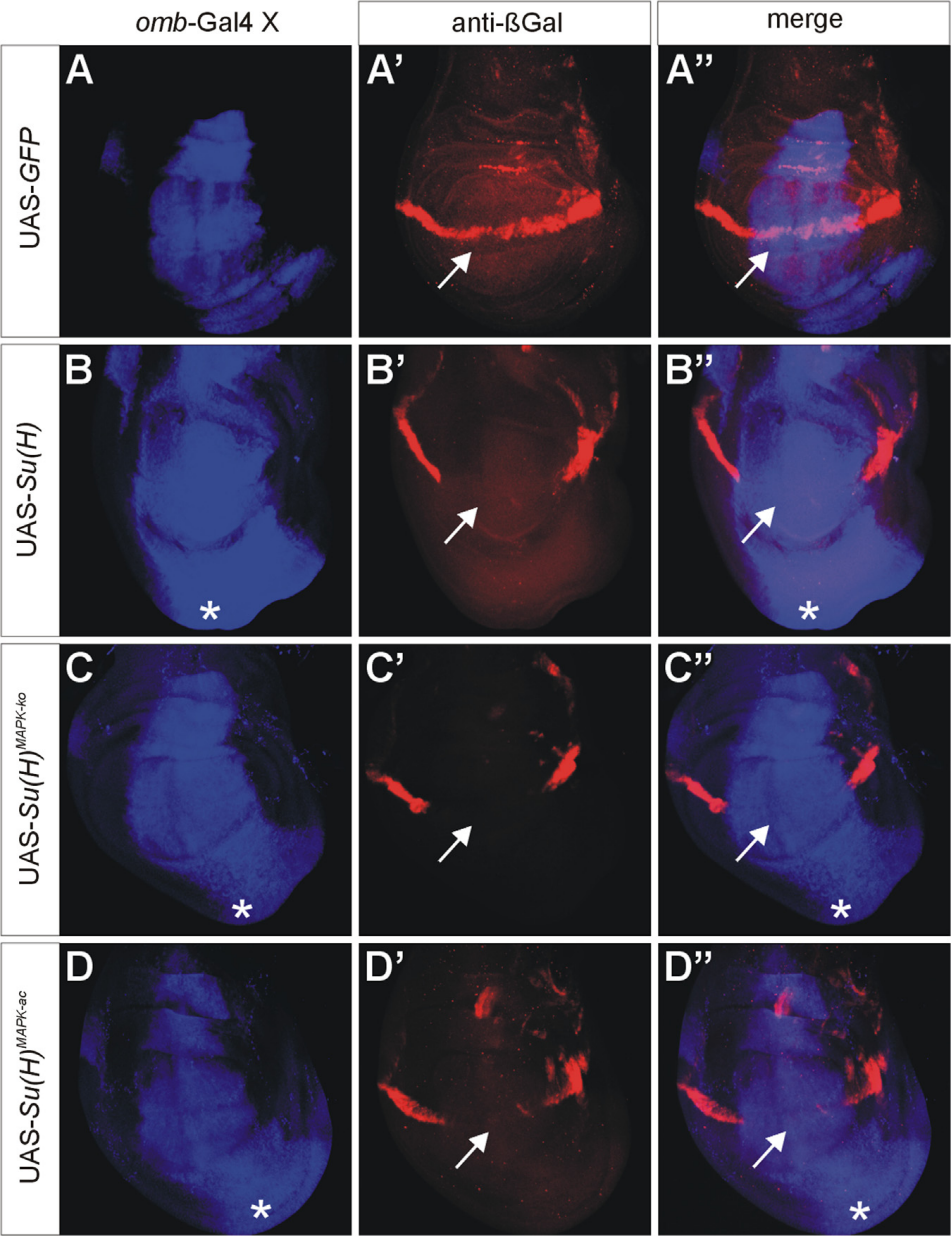
Response of the Notch target gene *E*(*spl*)*m8-HLH*. Overexpression of the UAS-*Su*(*H*) variants as indicated; the *omb*-expression domain is highlighted in blue in A–D and A″–D″ (A,A′ green fluorescent protein GFP; B–D and B′–D′, anti-Su(H) staining). Expression of the *E*(*spl*)*m8-HLH* reporter *E*(*spl*)*m8-lacZ* is shown in red (A′–D″). UAS-*GFP* served as control. Note repression of *E*(*spl*)*m8-lacZ* along the dorso-ventral boundary (arrows) upon overexpression of the three *Su*(*H*) variants (B′–D″); overgrowth of the ventral disc is marked by asterisks (B–D, B″–D″).

UAS-*Su*(*H*), UAS-*Su*(*H*)^*MAPK-*^^*ko*^ and UAS-*Su*(*H*)^*MAPK-ac*^ were overexpressed with *omb*-Gal4 [7] in wing imaginal discs of third instar *Drosophila* larvae. A total of four Notch target genes was analysed, *wingless* (Fig. 1) [8], *cut* (Fig. 2) [9], *E*(*spl*)*m8-HLH* [10] (using *E*(*spl*)*m8-lacZ* [11], Fig. 3) and *vestigial* [12] (using *vg*^*BE*^*-lacZ* [13], Fig. 4). Overall, overexpression of *Su*(*H*)^*MAPK-*^^*ko*^ caused a stronger response of the Notch target genes than that of wild-type *Su*(*H*), whereas *Su*(*H*)^*MAPK-ac*^ elicited the weakest effects, in agreement with a downregulation of *Su*(*H*) activity by MAPK-mediated phosphorylation [3].

### 1.2 Response of *cut* expression to the combined induction of *Su*(*H*) variants and activated components of the EGFR pathway during wing development

**Fig. 5:**
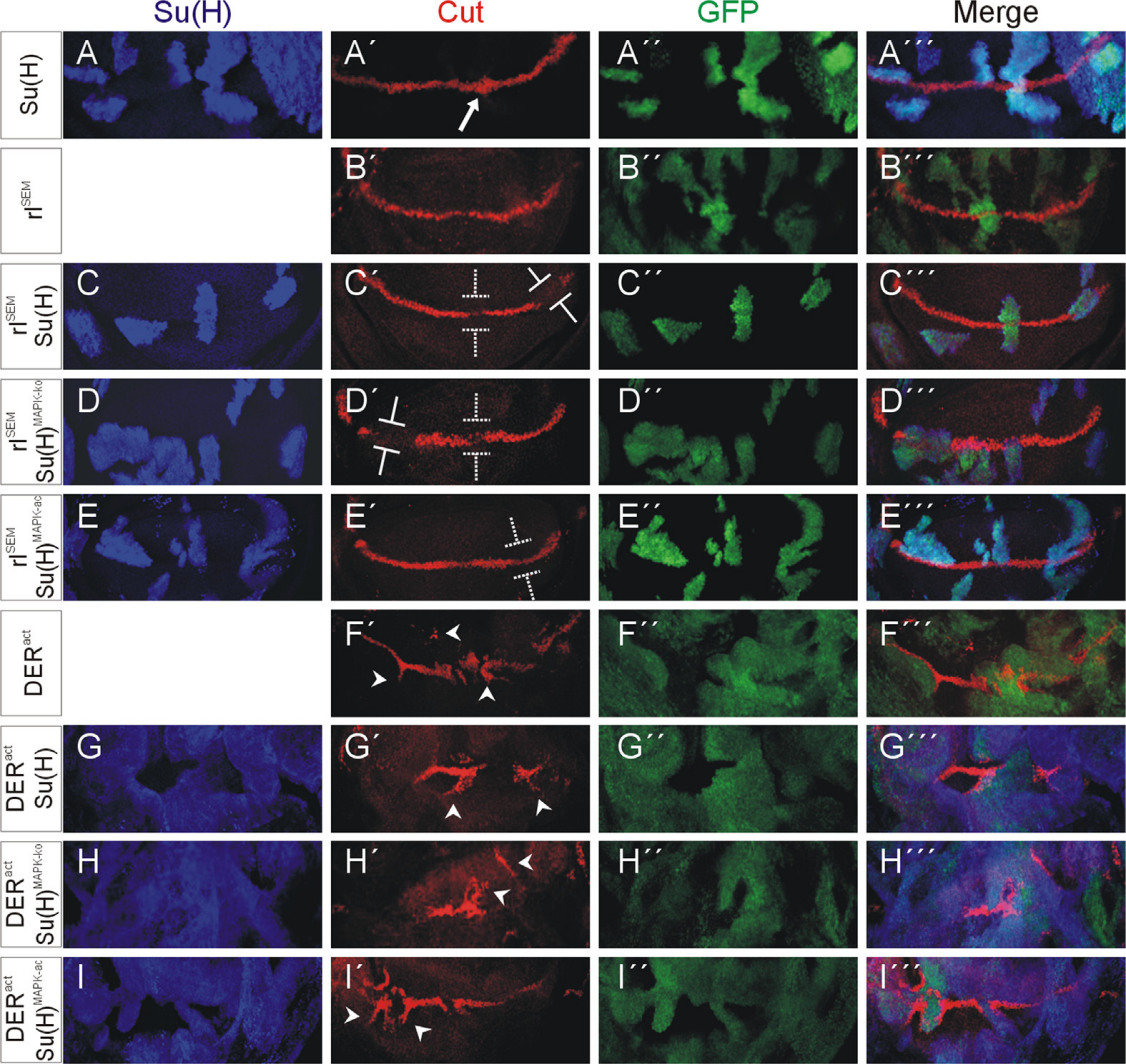
Expression of *cut* in response to *Su*(*H*), *DER*^*act*^ and *rl*^*SEM*^ overexpression. Overexpression clones were induced in wing imaginal discs. They are labelled by the presence of GFP (green in A″–I‴). Ectopic Su(H) protein is labelled in blue (A–I, A‴–I‴), and *cut* expression is shown in red (A′–I′ and A‴–I‴). Constructs indicated at the left were under UAS-control. Note induction of *cut* upon overexpression of *Su*(*H*) (arrow in A′), but repression of *cut* by simultaneous overexpression of *rl*^*SEM*^ (C′) labelled with blunt arrows. Likewise repression was seen in the combination with *Su*(*H*)^*MAPK-*^*^ko^* (D′) but not or weakly in combination with *Su*(*H*)^*MAPK-ac*^ (E’). *DER*^*act*^ overexpression clones were frequently distorted and induced *cut* expression along the boundary to the non-overexpressing cells (arrowheads in F′–I′).

The expression of the Notch target gene *cut* was analysed in cell clones overexpressing either of the three Su(H) isoforms alone or in combination with the activated EGF-receptor (*DER*^*act*^) or the activated MAPK (*rl*^*SEM*^) [14], [15] (Fig. 5). Overexpression clones were induced in wing imaginal discs [16]. *Su*(*H*) overexpression induced *cut* expression, whereas it repressed it when simultaneously overexpressed with *rl*^*SEM*^ (Fig. 5A–A‴ and C–C‴) [3]. Likewise repression was observed with *Su*(*H*)^*MAPK-*^^*ko*^, but less with *Su*(*H*)^*MAPK-ac*^ (Fig. 5D and E‴). Cell clones overexpressing *DER*^*act*^ were frequently distorted, and *cut* expression was induced at the boundary of *DER*^*act*^ expressing and non-expressing cells independent of the overexpression of any *Su*(*H*) variant (arrowheads in Fig. 5F′–I‴).

### 1.3 Adult phenotypes resulting from the combined overexpression of *Su*(*H*) variants and activated components of the EGFR pathway

**Fig. 6:**
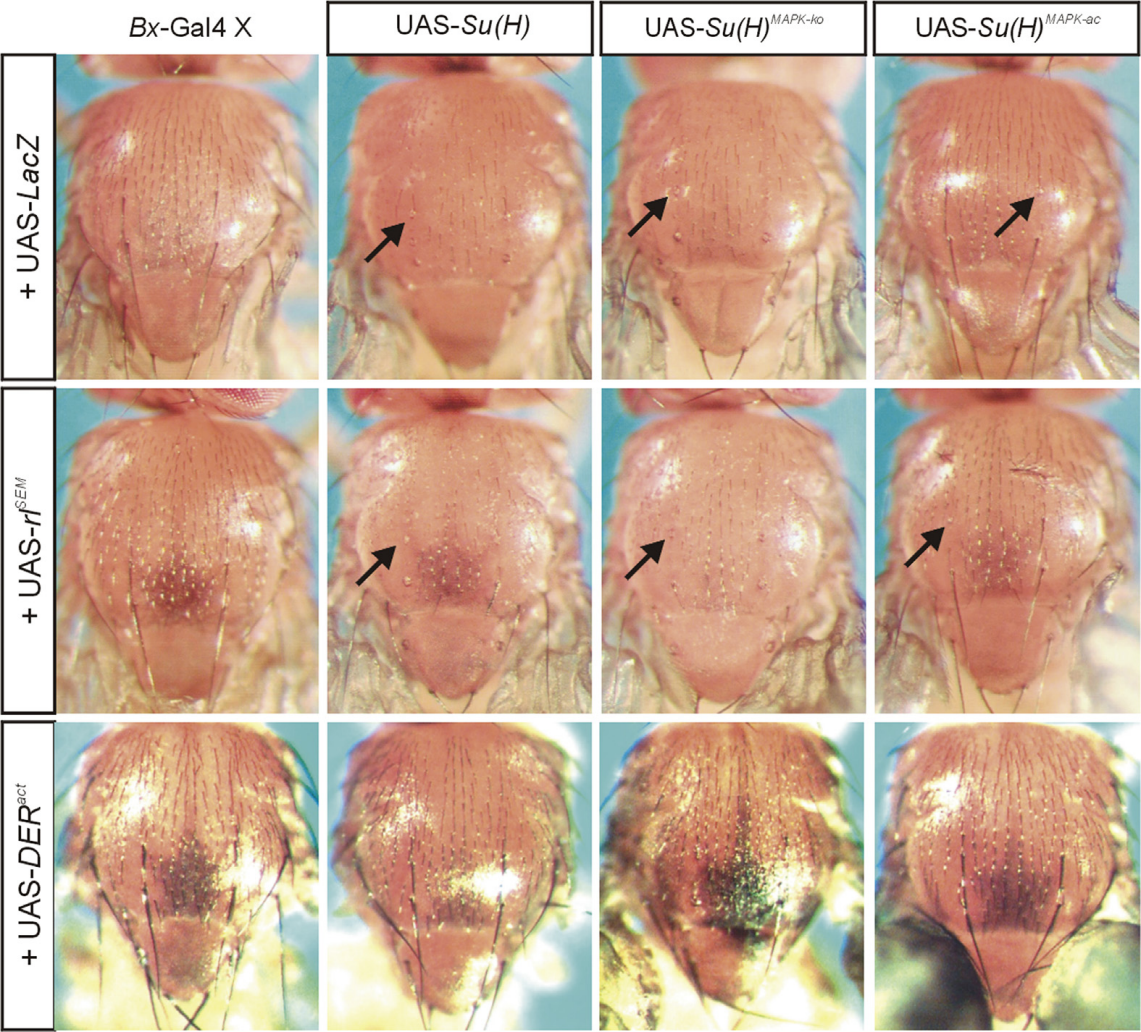
Overexpression consequences of *Su*(*H*), *DER*^*act*^ and *rl*^*SEM*^ during thorax development. Co-overexpression of UAS-*Su*(*H*) variants together with UAS-*lacZ* (control), UAS-*rl*^*SEM*^ or UAS-*DER*^*act*^ was driven in the developing thorax using *Bx*-Gal4 at 18 °C. Arrows point to examples of shaft to socket transformations that affected the majority of macrochaetae when UAS-*Su*(*H*) or UAS-*Su*(*H*)^*MAPK-ko*^ were overexpressed, but were rarely observed upon UAS-*Su*(*H*)^*MAPK-ac*^ ectopic expression. Simultaneous overexpression of UAS-*rl*^*SEM*^ had little influence on each of these specific phenotypes. In contrast UAS-*DER*^*act*^ phenotypes were epistatic to the overexpression of any the respective Su(H) constructs, i.e. all the resultant flies resembled those of the single *DER*^*act*^ overexpression. Typical representatives are shown in each case.

**Fig. 7:**
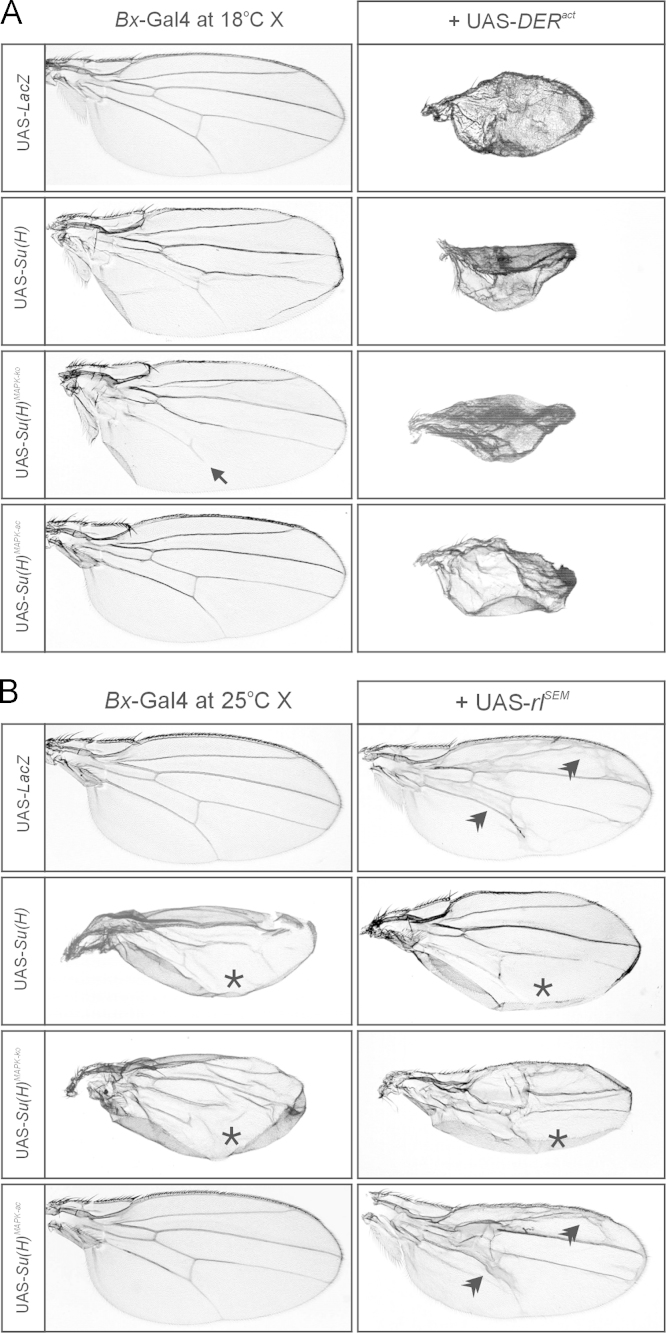
Overexpression consequences of *Su*(*H*), *DER*^*act*^ and *rl*^*SEM*^ during wing development. Co-overexpression of UAS-*Su*(*H*) variants together with UAS-*lacZ* (control), UAS-*DER*^*act*^ (at 18 °C) (A) or UAS-*rl*^*SEM*^ (at 25 °C) (B) was driven in the developing wing using *Bx*-Gal4. (A) At 18 °C, *Su*(*H*)^*MAPK-ko*^ repressed vein formation (arrow) which was not observed for either *Su*(*H*) or *Su*(*H*)^*MAPK-ac*^. Induction of UAS-*DER*^*act*^ resulted in very small wings mainly consisting of vein material, which was independent of *Su*(*H*) overexpression. As a consequence, the wings resulting from the combined overexpression were indistinguishable from those of the single *DER*^*act*^ overexpression. (B) At 25 °C, overexpression of either *Su*(*H*) or *Su*(*H*)^*MAPK-ko*^ but not *Su*(*H*)^*MAPK-ac*^ induced tissue overgrowth typified by wing blisters (asterisks). Induction of UAS-*rl*^*SEM*^ caused a network of veins (double arrowheads) which was repressed by the presence of ectopic *Su*(*H*) or *Su*(*H*)^*MAPK-ko*^ but not by *Su*(*H*)^*MAPK-ac*^. At the same time *Su*(*H*) and *Su*(*H*)^*MAPK-ko*^ gain of function phenotypes prevailed. Typical representatives are shown in each case.

**Fig. 8:**
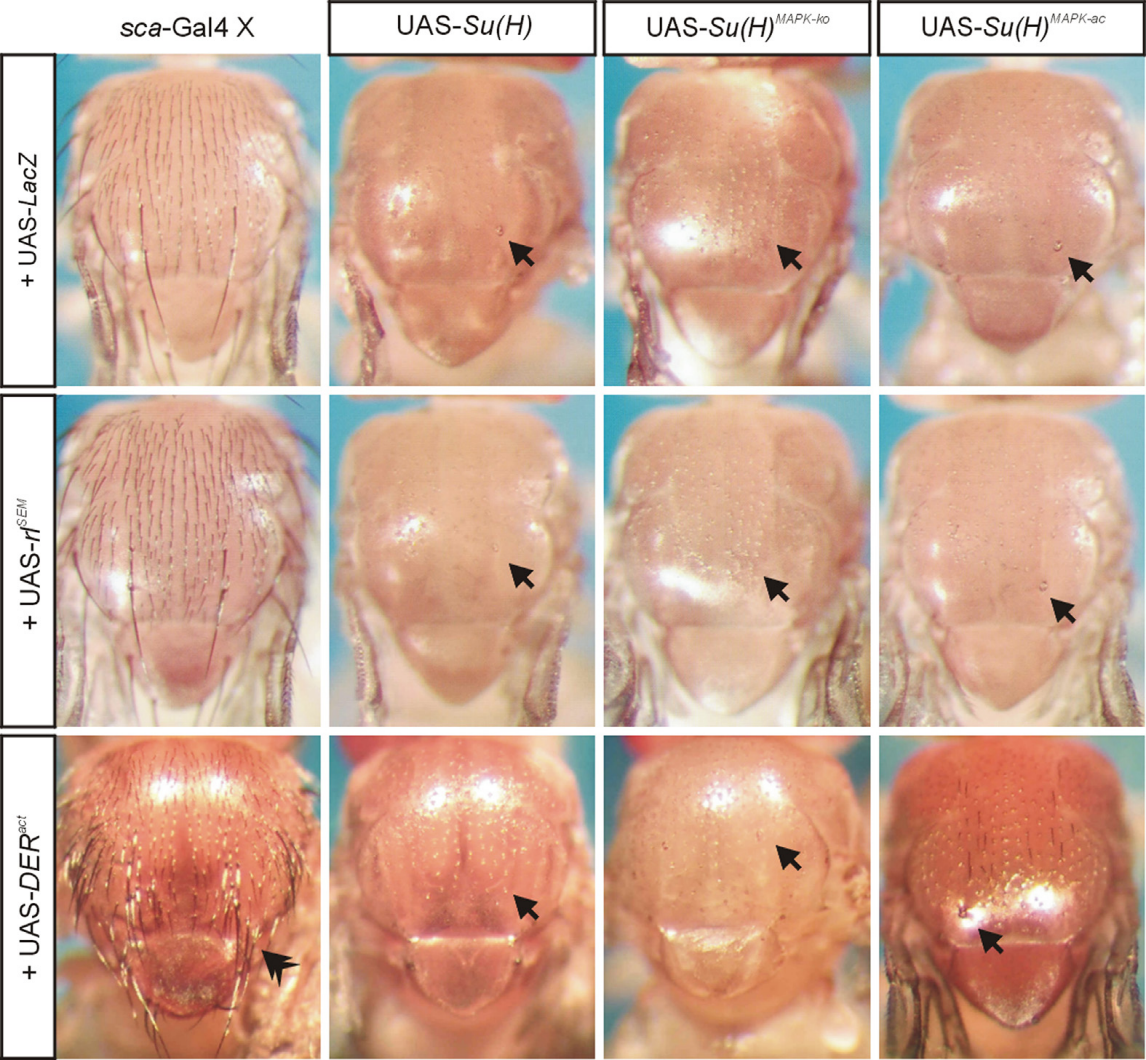
Overexpression consequences of *Su*(*H*), *DER*^*act*^ and *rl*^*SEM*^ in the developing bristle organs. Co-overexpression of UAS-*Su*(*H*) variants together with UAS-*lacZ* (control), UAS-*rl*^*SEM*^ or UAS-*DER*^*act*^ was driven in the developing bristle organs using *sca*-Gal4 at 25 °C. Overexpression of any of the *Su*(*H*) variants within the developing bristle organ caused a near complete transformation of bristle shafts to sockets of micro- or macrochaetae. Examples of the resultant double sockets are highlighted by arrows. The phenotypes were nearly indistinguishable between the three Su(H) variants. Whereas flies overexpressing of *sca*::*rl*^*SEM*^ matched the control phenotype, *sca*:: *DER*^*act*^ developed tufts of macrochaetae on the posterior thorax (double arrowhead). Each of these phenotypes disappeared completely in a combination with any *Su*(*H*) variant. Typical representatives are shown in each case.

**Fig. 9:**
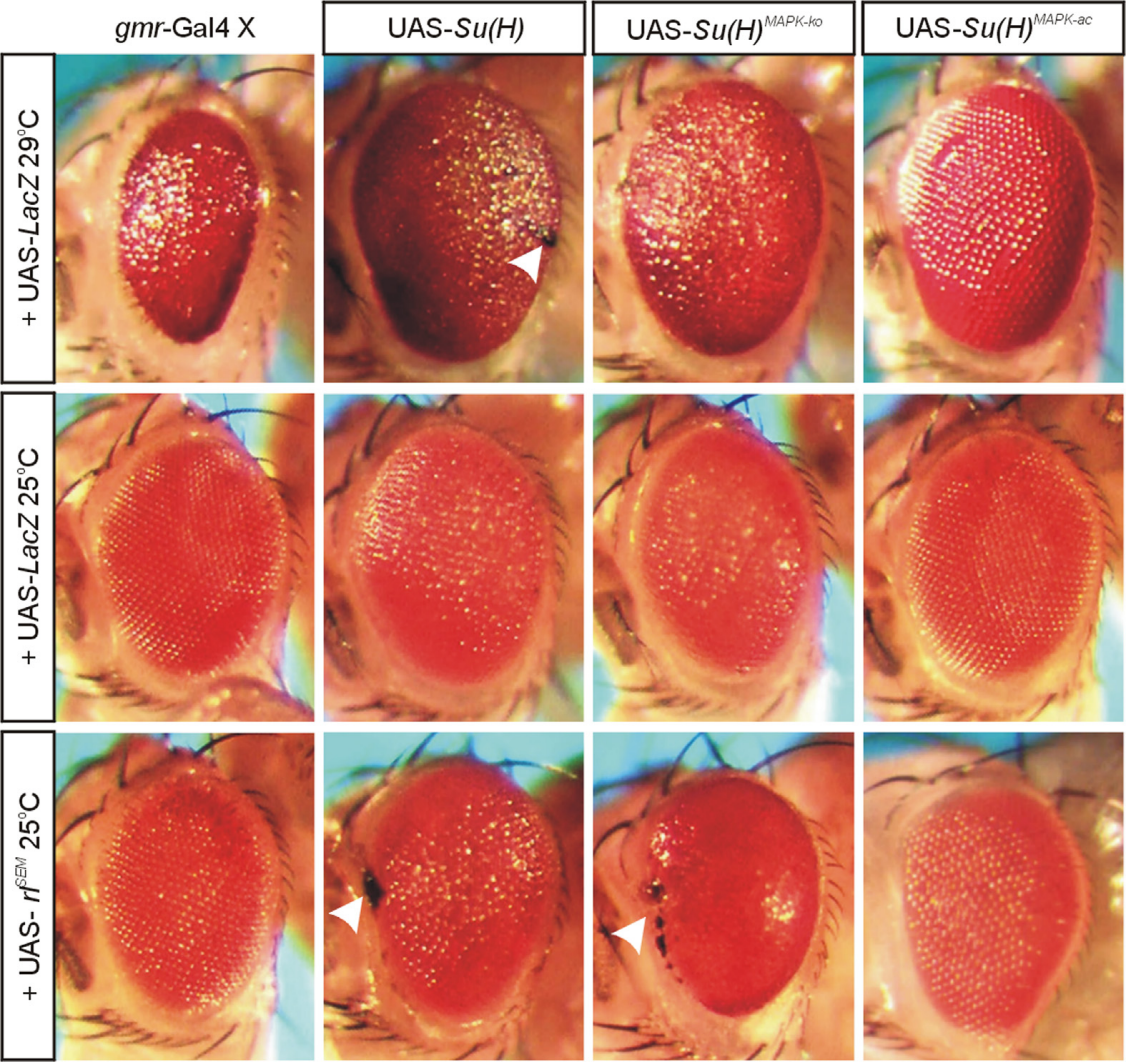
Overexpression consequences of *Su*(*H*), *DER*^*act*^ and *rl*^*SEM*^ in the developing eye. Co-overexpression of UAS-*Su*(*H*) variants together with UAS-lacZ (control) or UAS-*rl*^*SEM*^ was driven in the developing eye using *gmr*-Gal4. At 29 °C, *gmr*::lacZ flies have smaller eyes with irregular facets giving the eye a rough appearance. In contrast, overexpression of *Su*(*H*) variants at this temperature causes enlarged eyes that appear slightly bulgy. Both *Su*(*H*) and *Su*(*H*)^*MAPK-ko*^ induced irregularities in the arrangement of the facets and necrosis (arrowhead), in contrast to *Su*(*H*)^*MAPK-ac*^. At 25 °C the phenotypes are much milder, and eyes appear like wild type (*Su*(*H*)^*MAPK-ac*^) or slightly rough (*Su*(*H*) and *Su*(*H*)^*MAPK-ko*^). A similar rough eye phenotype was observed upon induction of *rl*^*SEM*^ at 25 °C. The combined overexpression of *Su*(*H*) and *rl*^*SEM*^ gave a mixed phenotype, i.e. eyes were smaller, rough and necrotic (arrowhead). Similar necrotic patches (arrowhead) and size decrease were also observed in the eyes of *gmr*:: *Su*(*H*)^*MAPK-ko*^+*rl*^*SEM*^ animals, which in addition had a glossy appearance. In contrast, the eyes of the *gmr*:: *Su*(*H*)^*MAPK-ac*^+*rl*^*SEM*^ animals looked similar to *gmr*:: *rl*^*SEM*^. Typical representatives are shown in each case.

Overexpression of UAS-*DER*^*act*^ in the thorax (Fig. 6) or the wing anlagen (Fig. 7A) using *Bx*-Gal4 [17] was fully epistatic to the *Su*(*H*) gain of function phenotypes. This was in contrast to the simultaneous overexpression of UAS-*rl*^*SEM*^ with the UAS-*Su*(*H*) isoforms: in these experiments the *Su*(*H*) gain of function phenotypes prevailed (Figs. 6 and 7B). It has been described before that the overexpression of *Su*(*H*) in the developing sensory organs using *sca*-Gal4 causes a shaft to socket transformation [18], which we also observed upon overexpression of *Su*(*H*)^*MAPK-*^^*ko*^ or *Su*(*H*)^*MAPK-ac*^ (Fig. 8). Whereas *sca*::*rl*^*SEM*^ was similar to the control, *sca*:: *DER*^*act*^ animals developed tufts of macrochaetae on the posterior thorax (Fig. 8). Interestingly, in combination with any of the *Su*(*H*) variants, the double socket phenotype resulting from *Su*(*H*) overexpression prevailed (Fig. 8). Finally, consequences of *Su*(*H*) overexpression in the developing eye using *gmr*-Gal4 were addressed (Fig. 9). As the Gal4::UAS system is temperature sensitive, phenotypes were strong at 29 °C, revealing defects in the control as well [19]. At this temperature, *Su*(*H*) overexpression caused overgrowth of the eye, irregular facets and necrosis. At 25 °C the phenotypes were much weaker resembling the control. A combination with *rl*^*SEM*^ enhanced the irregular facet and necrotic phenotype, whilst *gmr*::*rl*^*SEM*^ flies were very similar to the control (Fig. 9).

## 2 Experimental design, materials and methods

### 2.1 Fly stocks, husbandry and analyses

Flies were obtained from the Bloomington stock collection if not noted otherwise. Fly husbandry was according to standard protocols at 29 °C, 25 °C or 18 °C as noted. *y*^*1*^ *w*^*1118*^, UAS-*lacZ* and UAS-*GFP* served as control. For information on fly stocks we refer to http://flybase.bio.indiana.edu. Adult wings of female flies were dehydrated in ethanol and mounted in Euparal (Roth, Karlsruhe, Germany) and dried over night. Pictures of wings or adult flies were taken on a Zeiss Axiophot or a Wild 5M stereomicroscope, respectively, using an ES120 camera (Optronics, Goleta CA, USA) and Pixera Viewfinder software, version 2.0.

Generation of UAS-*Su*(*H*), UAS-*Su*(*H*)^*MAPK-ko*^ (T426A) and UAS-*Su*(*H*)^*MAPK-ac*^ (T426E) was described earlier [3], [20]. UAS-*rl*^*SEM*^ was provided by Martín-Blanco [15] and UAS-*DER*^*act*^ by Freeman [14]. LacZ-reporter gene constructs *vg*^*BE*^*-lacZ* and *E*(*spl*)*m8-lacZ* were kindly provided by Bray and Schweisguth [11], [13]. Tissue-specific expression of respective transgenes was induced with the Gal4:: UAS-system [6] using *omb*-Gal4 [7], *Bx*-Gal4 [17], *sca*-Gal4 [21] and *gmr*-Gal4 [19]. Overexpression clones were induced by the flip-out technique [16] with the following fly lines: *y w* flp^1.22^; UAS-*Su*(*H*) or UAS-*Su*(*H*) mutants, *y w* flp^1.22^; UAS-*rl*^*SEM*^ and *y w* flp^1.22^; UAS-*rl*^*SEM*^ UAS-*Su*(*H*); UAS-*DER*^*act*^ and *y w* flp^1.22^; UAS-*DER*^*act*^ UAS-*Su*(*H*) or UAS-*Su*(*H*) mutants and *y w* Act>CD2>Gal4, UAS-*GFP*-nls (kindly provided by K. Basler).

### 2.2 Immunohistochemistry

Imaginal discs were stained according to standard protocols using mouse monoclonal antibodies directed against Cut, Wingless or beta-Galactosidase (developed by G.M. Rubin, S.M. Cohen, and J.R. Sanes respectively, and obtained from DSHB or using a polyclonal antiserum directed against Su(H)) [22]. Secondary antibodies coupled to FITC, Cy3 or Cy5 (1:200) were obtained from Jackson Immuno-Research Laboratories (Dianova, Hamburg, Germany). Samples were mounted in Vectashield (Vector Lab) and examined on a Zeiss Axioskop coupled to a BioRad MRC1024 confocal microscope using LaserSharp 2000TM software (Carl Zeiss, Jena, Germany).
